# Triboelectric Response of Electrospun Stratified PVDF and PA Structures

**DOI:** 10.3390/nano12030349

**Published:** 2022-01-22

**Authors:** Pavel Tofel, Klára Částková, David Říha, Dinara Sobola, Nikola Papež, Jaroslav Kaštyl, Ştefan Ţălu, Zdeněk Hadaš

**Affiliations:** 1 Department of Physics, Faculty of Electrical Engineering and Communication, Brno University of Technology, Technická 2848/8, 616 00 Brno, Czech Republic; tofel@vut.cz (P.T.); xrihad01@vutbr.cz (D.Ř.); sobola@vut.cz (D.S.); papez@vut.cz (N.P.); 2 Central European Institute of Technology, Purkyňova 656/123, 612 00 Brno, Czech Republic; klara.castkova@ceitec.vutbr.cz (K.Č.); jaroslav.kastyl@ceitec.vutbr.cz (J.K.); 3 Department of Ceramics and Polymers, Faculty of Mechanical Engineering, Brno University of Technology, Technická 2896/2, 616 69 Brno, Czech Republic; 4 Institute of Physics of Materials, Czech Academy of Sciences, Žižkova 22, 616 62 Brno, Czech Republic; 5 Department of Inorganic Chemistry and Chemical Ecology, Dagestan State University, St. M. Gadjieva 43-a, 367015 Makhachkala, Russia; 6 Directorate of Research, Development and Innovation Management (DMCDI), Technical University of Cluj-Napoca, Constantin Daicoviciu Street, No. 15, 400020 Cluj-Napoca, Romania; 7 Institute of Solid Mechanics, Mechatronics and Biomechanics, Faculty of Mechanical Engineering, Brno University of Technology, Technická 2896/2, 616 69 Brno, Czech Republic; Zdenek.Hadas@vut.cz

**Keywords:** dielectric properties, electrospinning, fiber composite, PVDF, PA, TENG, triboelectric effect

## Abstract

Utilizing the triboelectric effect of the fibrous structure, a very low cost and straightforward sensor or an energy harvester can be obtained. A device of this kind can be flexible and, moreover, it can exhibit a better output performance than a device based on the piezoelectric effect. This study is concerned with comparing the properties of triboelectric devices prepared from polyvinylidene fluoride (PVDF) fibers, polyamide 6 (PA) fibers, and fibrous structures consisting of a combination of these two materials. Four types of fibrous structures were prepared, and then their potential for use in triboelectric devices was tested. Namely, individual fibrous mats of (*i*) PVDF and (*ii*) PA fibers, and their combination—(*iii*) PVDF and PA fibers intertwined together. Finally, the fourth kind was (*iv*), a stratified three-layer structure, where the middle layer from PVDF and PA intertwined fibers was covered by PVDF fibrous layer on one side and by PA fibrous layer on the opposite side. Dielectric properties were examined and the triboelectric response was investigated in a simple triboelectric nanogenerator (TENG) of individual or combined (*i–iv*) fibrous structures. The highest triboelectric output voltage was observed for the stratified three-layer structure (the structure of *iv* type) consisting of PVDF and PA individual and intertwined fibrous layers. This TENG generated 3.5 V at peak of amplitude at 6 Hz of excitation frequency and was most sensitive at the excitation signal. The second highest triboelectric response was observed for the individual PVDF fibrous mat, generating 2.8 V at peak at the same excitation frequency. The uniqueness of this work lies in the dielectric and triboelectric evaluation of the fibrous structures, where the materials PA and PVDF were electrospun simultaneously with two needles and thus created a fibrous composite. The structures showed a more effective triboelectric response compared to the fibrous structure electrospun by one needle.

## 1. Introduction

Energy harvesters and sensors based on the triboelectric effect have been a very promising research field in the last ten years. Their advantage is in a wide range of materials that can be used and the simple fabrication process of these triboelectric devices. Many researchers work on different kinds of devices using the triboelectric effect. This effect can be used in devices such as self-powered textiles [1], wearable electronics [2], self-powered human motion sensors [3], self-powered automobile sensors [4], position sensing and touchpads [5], flexible nano generators [6], non-invasive biomedical monitoring systems [7], energy harvesters for devices in internet of things infrastructure [8], environmental monitoring systems [9], air filters [10], and topically very important research into protection against a coronavirus pandemic, where the simple triboelectric nanogenerator with an electrocution layer may serve the purpose of filtration and the deactivation of SARS-CoV-2 [11]. These perspectives lead us to study this phenomenon and utilize its potential in simple devices based on a fibrous structure. From a triboelectric series, a combination of PVDF (polyvinylidene fluoride) and PA (polyamide 6) creates a very effective triboelectric device [12].

**Figure 1:**
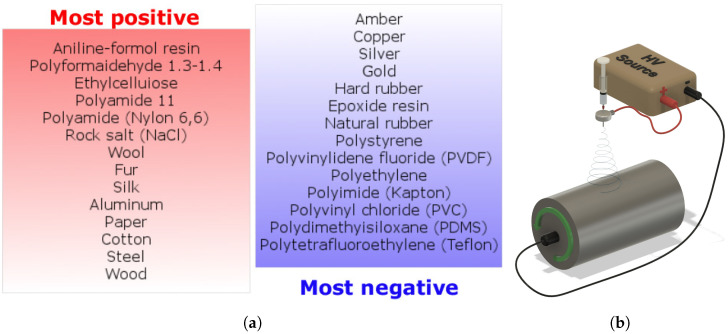
(**a**) A triboelectric series assembled from various works. (**b**) Scheme of electrospinning process.

The triboelectric effect and level of contact electrification performance are based on materials that come into contact. In this case, contact electrification occurs caused by a surface transference of electrons or ions between these two materials [13]. Each material has its own ability to lose or gain electrons during the contact electrification process. This ability can be found in a list of materials (triboelectric series) where polarity and amount of charge for each material are described. An example of the triboelectric series can be seen in Figure 1a (based on various works [14,15,16,17]). PVDF is a material which tends to gain electrons during electrification. On the other hand, PA is positively charged during electrification. Both materials lie far enough apart in the triboelectric series, and this distance indicates their high triboelectric potential on contact.

One of the most used fabrication techniques for the preparation of fibrous structures, which can be used as tribomaterials, is electrospinning [18,19,20]. During the electrospinning, a polymer solution is ejected from a needle tip by applying a high voltage between the needle and a grounded collector, as illustrated in Figure 1b. When an electrostatic force overcomes the surface tension at the tip of the needle, a so-called Taylor’s cone is formed, and it is elongated into a fluid jet. The jetting fluid is collected on a collector in the form of fibers due to the potential difference between the needle tip and the collector. The electrospinning process produces material in the form of long and thin nanofibers [18,21]. The electrospun material has a rough surface and a significantly larger surface area per unit volume than the material prepared as a bulk [22], which can be highly beneficial in applications such as triboelectric devices. Fibers significantly improve the triboelectric effect of electrospun materials, as their large surface area allows the generation of a large number of charges during electrification [23,24,25].

Electrospinning is often used to prepare materials where it is necessary to increase their surface area [26,27]. Mostly are reported the combinations of electrospun nanofibrous mats as one triboelectric layer against another triboelectric layer which is in solid-state [28], porous form [25], or in the form of some nanostructure [29]. All these modifications lead to enhancing the triboelectric output performance of the triboelectric device. The PVDF can be prepared in polarized and unpolarized forms, but no significant difference was reported between these two forms if the PVDF is used as a triboelectric device [23,26].

It is very interesting to study multilayer triboelectric devices, where an interlayer is situated between tribomaterial and electrode. This interlayer is used for charge-trapping and significantly increases the triboelectric output [26]. It has been demonstrated that triboelectric device assembled from PVDF cast on polyimide increased the triboelectric output 8× [30]. If the polydimethylsiloxane (PDMS) is used instead of polyimide, the triboelectric output can increase even more [31].

Our research into the triboelectric devices was focused on electrospun materials such as PVDF and PA, and their combinations in fiber form with the effort to evaluate their possibilities and performance in triboelectric devices. These materials are widely used in triboelectric devices for their non-reactivity, mechanical robustness, and flexibility [32]. Different combinations of tribomaterials were fabricated from these nanofiber materials, which were consequently tested from the triboelectric point of view. Only a few researchers are focused on the triboelectric properties of the fiber materials where two materials are intertwined between each other. For example, Garcia et al. present work where two fiber materials were sandwiched between electrodes, and this device can be used as a simple self-powered pressure sensor [33]. The fibrous materials in this work were prepared separately and then compressed between two electrodes. The PVDF and PA fibrous materials were electrospun individually or simultaneously using a two-needle setting. In order to study the triboelectric performance, the fibrous materials were arranged in different types of simple or stratified devices and characterized.

### The V-Q-x Relationship for Contact-Separation Mode

**Figure 2:**
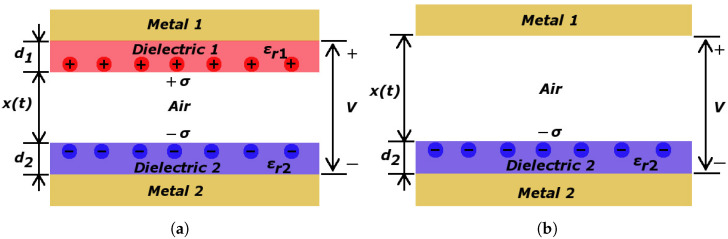
Contact-separation mode theoretical model. (**a**) Dielectric-to-dielectric and (**b**) conductor-to-dielectric.

The triboelectric nanogenerator (TENG) generally operates in one of four basic modes such as contact-separation (CS) mode, lateral sliding (LS) mode, single-electrode (SE) mode, and freestanding triboelectric-layer (FT) mode [34]. We have focused on CS mode because TENG is simple to manufacture and is very widely used. TENGs are commonly described by the V-Q-x relationship, where *V* represents the voltage between electrodes, *Q* is the amount of charge transferred between the electrodes, and *x* is a separation distance of the electrodes. The theoretical model for the contact separation mode is shown in Figure 2. Figure 2a represents TENG, where two dielectric materials are used, while Figure 2b represents TENG with one dielectric material.

The output voltage of TENG in contact mode can be expressed by V-Q-x relationship [35,36,37]:

(1) $$V=-\frac{Q}{S\varepsilon_{0}}\left(d_{0}+x\left(t\right)\right)+\frac{\sigma x(t)}{\varepsilon_{0}},$$

where *Q* is the charge, *S* is the area, $\varepsilon_{0}$ is the permittivity of space, x(t) is the displacement of electrodes as a function of time, σ is the triboelectric charge density, and $d_{0}$ is given:

(2) $$d_{0}=\frac{d_{1}}{\varepsilon_{r}1}+\frac{d_{2}}{\varepsilon_{r}2}.$$

At open-circuit, the conditions are no charge transferred, and *Q* is equal to zero. Then open-circuit voltage $V_{\mathrm{oc}}$ is given:

(3) $$V_{\mathrm{oc}}=\frac{\sigma x(t)}{\varepsilon_{0}}.$$

On the contrary, at short circuit conditions, *V* is equal to zero. Therefore, a short circuit current $I_{\mathrm{sc}}$ is given:

(4) $$I_{\mathrm{sc}}=\frac{S\sigma d_{0}v\left(t\right)}{{\left(d_{0}+x\left(t\right)\right)}^{2}}.$$

$V_{\mathrm{oc}}$ and $I_{\mathrm{sc}}$ are dependent on the triboelectric charge density σ. The charge density σ and space distance *x* influences $V_{\mathrm{oc}}$, whereas the $I_{\mathrm{sc}}$ is further dependent on the contact speed [35,36,37]. Thus it can be concluded that the materials in the triboelectric devices can be compared in terms of $V_{\mathrm{oc}}$ values, where the speed of contact is ignored.

## 2. Material and Methods

The electrospun polyvinylidene fluoride (molar mass 275,000 g/mol, Sigma Aldrich, St. Louis, MO, USA) and polyamide 6 (molar mass 35,000 g/mol, Alfa Chemicals, Bracknell, UK) were used for electrospinning. Dimethylsulfoxide p.a. (DMSO, Sigma Aldrich), acetone p.a. (Ac, Sigma Aldrich), acetic acid (AA, Penta, Bratislava, Slovakia), and formic acid (FA, Merck, Darmstadt, Germany) were used for polymers solutions preparation [18,19,20,38,39]. For PVDF solutions, solvents DMSO and Ac were mixed in a volume ratio 7:3. The PVDF beads were dissolved in the binary solvent in a concentration of 20 wt% at 50 °C for 24 h until a visually homogeneous solution was formed. For PA solutions, solvents AA and FA were mixed in a volume ratio 8:2. The PA granules were dissolved in the binary mixture in a concentration of 15 wt% at lab temperature for 24 h until a clear solution was achieved.

The prepared solutions were electrospun using the 4SPIN electrospinning equipment (Contipro, Czech Republic) at a feeding rate of (10–20) μL/min through a needle. Needle diameter and rotation speed for each structure are shown in Table 1. The accelerating voltage was 40 kV, and the distance between the needle tip and the collector (rotating cylinder covered by aluminum foil) was 15 cm. These electrospinning parameters were constant for all structures. The fibrous samples PVDF and PA were collected for 90 min in the form of non-woven structures, which were further characterized.

A special arrangement was used for samples PVDF+PA and S(PVDF+PA). The combined spinning (co-spinning) using two independent needles with separate feeding by PVDF and PA was applied for 90 min to spin sample PVDF+PA with intertwining fibers. In the case of S(PVDF+PA) sample, the structure was spun layer by layer. Firstly, the PVDF layer was spun for 45 min, then PVDF+PA layer co-spun for 45 min and, finally, the PA layer was spun for 45 min. See Table 1 for the details of samples and processing parameters.

The final thickness of our samples was measured by interferometer ILD 1402-10 (Micro Epsilon, Ortenburg, Germany). A thin metal plate in the shape of a square with 30 mm edge and a thickness of 0.1 mm was placed on the final fibrous structure, which was still on the Al foil. The thickness was then measured using an ILD 1402-10 at the center of the plate. After subtracting the thickness of the Al foil and the metal plate, the thickness of the fibrous structure was obtained.

Dielectric properties were measured by Alpha-A High Performance Modular Measurement System (Novocontrol, Montabaur, Germany). As a sample holder, the 16451B dielectric test fixture (Agilent, Tokyo, Japan) with a dimension of the active electrode 5 mm was used. The triboelectric energy performance of the prepared samples was evaluated by electrometer 6517b (Keithley, Solon, OH, USA). The TENG was assembled in vertical contact-separation mode. The moving part consisting of the Cu electrode was controlled by the vibration test system TV 50018 (Tira, Schalkau, Germany). The sample was clamped on a fixed Cu electrode. The area of the active part of the generator was (30 × 30) mm. Mechanical force was measured by force sensor 208C01 (PCB Piezotronics, Hückelhoven, Germany), and this sensor was situated on the side of the fixed electrode. The displacement between electrodes was measured via interferometer ILD 1402-10 (Micro Epsilon, Ortenburg, Germany). Output voltages generated by the triboelectric devices were measured by oscilloscope DSOX2024A (Keysight, Santa Rosa, CA, USA). The same oscilloscope was used for the evaluation of distance between electrodes measured by interferometer.

Dynamic Signal Acquisition Module NI-9234 (National Instruments, Austin, TX, USA) was used for the force sensor, and the communication was performed via SignalExpress software. Measured data were processed by Matlab R2018a software.

**Figure 3:**
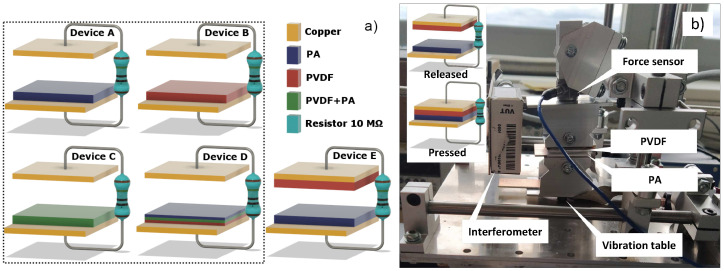
(**a**) Triboelectric devices assembled from different fibrous mats containing PVDF and PA material. The dashed line indicates triboelectric devices consisting of a single dielectric. (**b**) The measuring setup for triboelectric response measurement, where the inset figure illustrate pressed and released state of TENG during measurement.

The triboelectric materials were arranged into simple triboelectric devices operating in contact-separation mode. Five triboelectric devices were assembled where four devices were the type of conductor-to-dielectric, and one triboelectric device was the dielectric-to-dielectric type, as is shown in Figure 3a. The measurement setup is shown in Figure 3b, which also shows the pressed and released state of the triboelectric device during the measurement. The area of the triboelectric material in TENG was (30 × 30) mm. Device A used PA fibers against copper electrode, device B used PVDF fibers against copper electrode, device C was based on the triboelectric layer of PVDF+PA sample with intertwined fibers, and device D was compiled from the triple-layer dielectric fibrous structures. Device E was assembled with PVDF fibers on one electrode against PA fibers on the other electrode, creating a triboelectric device where the two dielectrics come into contact during operation. This device is presented here as an example of the standard use of these two materials in the TENG device to exploit their maximum triboelectric potential. We can compare how triboelectric devices with one dielectric against a metal electrode perform compared to this standard device E, where two dielectric materials come into contact.

An upper electrode was fixed, and the bottom electrode was controlled by a shaker. For this simple comparison, the devices were connected to an oscilloscope over 10 MΩ probe.

The quasi-static piezoelectric constant $d_{33}$ was measured using a Berlincourt $d_{33}$ meter (YE2730A, Sinocera, China) on electrospun structures sandwiched between two copper electrodes. All the prepared fibrous structures exhibited zero or negligible piezoelectricity with a maximum value of up to 1 pC/N.

## 3. Results and Discussion

**Figure 4:**
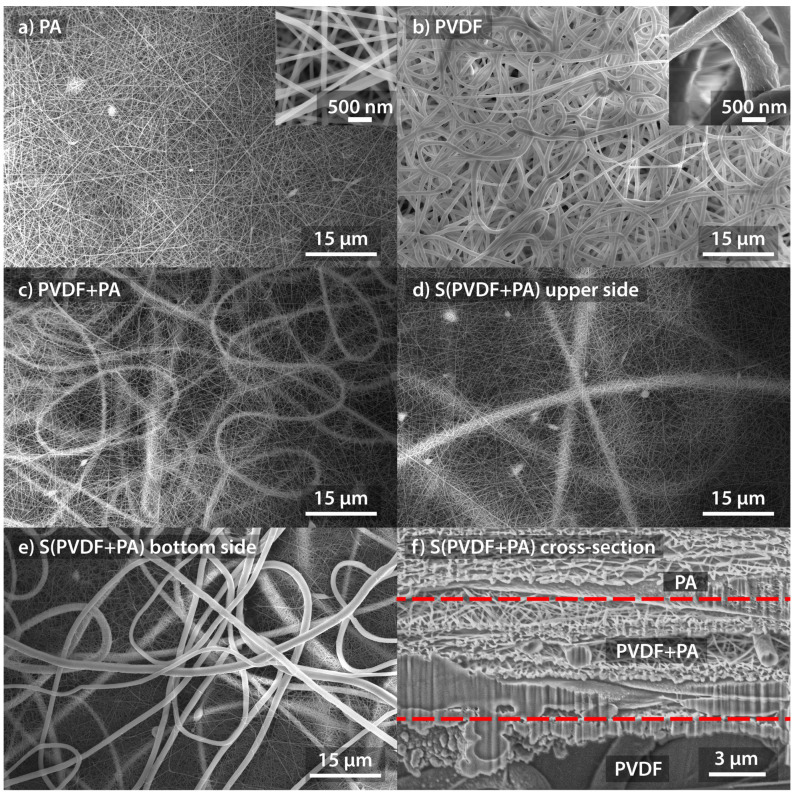
SEM images of (**a**) PA sample, (**b**) PVDF sample, (**c**) PVDF+PA sample, (**d**) top side of S(PVDF+PA) sample, (**e**) bottom side of S(PVDF+PA) sample (the side in contact with alumina foil during electrospinning process), and (**f**) cross-section of S(PVDF+PA) sample.

Four fibrous structures were prepared by electrospinning, and the resulting SEM images of individual samples are shown in Figure 4. The PA sample (Figure 4a) consisted of slim fibers with a diameter around 100 nm. On the contrary, the thick fibers can be observed for the PVDF sample (Figure 4b), where the fiber diameter was approx. 1000 nm. A combination of slim and thick fibers can be observed in Figure 4c, where the structure of PVDF+PA sample is depicted. The structure of the layered S(PVDF+PA) sample is shown in Figure 4d and Figure 4e, where the top and bottom sides are shown, respectively. The cross-section of the layered S(PVDF+PA) sample is shown in Figure 4f.

### 3.1. Dielectric Properties of Electrospun Samples

Materials prepared in a fibrous form basically exhibit lower dielectric constant against their bulky counterparts due to the high porosity content [40]. Measurement of the dielectric properties is challenging for the fiber materials because the measurement strongly depends on the air involved in the fiber mats structure. Nevertheless, if the pressure during measurement is of the same value for all measured samples, then the dielectric constant and other dielectric properties can be compared with each other.

**Figure 5:**
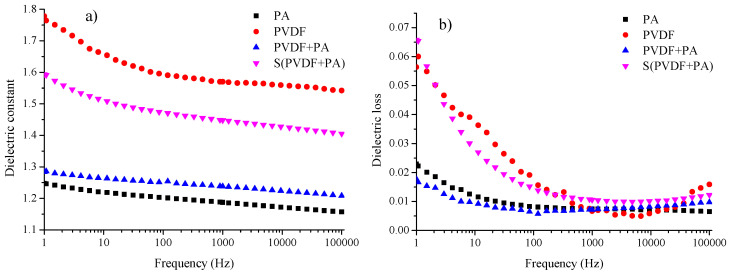
Dielectric properties of the prepared samples, (**a**) dielectric constant, and (**b**) dielectric loss in frequency dependence.

The dependence of the dielectric constant on frequency determined for prepared fibrous structures is shown in Figure 5a. The dielectric constant decreases with increasing frequency without any significant extremes for our samples, indicating our structures’ dielectric behavior. It is necessary to note that all fibrous materials were prepared at a final thickness of around 20 μm.

Generally, the PA has the dielectric constant of around 4 in the dense form [37,41]. Our PA sample exhibited the dielectric constant $\varepsilon_{r}=1.19$ at 1 kHz. The dielectric constant decreases almost linearly with increasing frequency. More significant reduction in dielectric constant was achieved for the sample formed by PVDF fibers. We have obtained the dielectric constant $\varepsilon_{r}=1.57$ at 1 kHz for the PVDF sample, which is roughly one-tenth of pure and dense PVDF material with α-phase (the β-phase slightly reduces this value) [16]. This is due to the high porosity of the sample, as it is made up of thick sparse fibers, as can be seen from Figure 4b. The combination of PVDF and PA fibers created a structure where the dielectric constant is around $\varepsilon_{r}=1.24$ at 1 kHz. The density of PVDF fibers is not large compared to PA fibers in the sample structure (see Figure 4c). Therefore, the dielectric constant’s dependence is more similar to that measured on PA fibers. The last layered sample S(PVDF+PA) further increased the dielectric constant on $\varepsilon_{r}=1.45$ at 1 kHz. The mentioned increase in the dielectric constant was due to the higher number of PVDF fibers in the S(PVDF+PA) sample compared to the PVDF+PA sample.

The dielectric losses versus frequency measured for the fabricated fibrous structures are shown in Figure 5b. The PA sample and the combination of PVDF and PA fibers (sample PVDF+PA) showed very low losses over the entire measured frequency spectrum. Both dependencies had a similar pattern and exhibited a dielectric loss of 0.7% at 1 kHz. Low losses were also observed at 1 Hz, where PA sample and the PVDF+PA sample exhibited dielectric loss 2.3% and 1.8%, respectively. On the other hand, the PVDF sample also exhibited low losses at 1 kHz (tan*δ* = 0.7%), but also high losses at low frequencies (tan*δ* = 6% at 1 Hz). Specified dielectric loss behavior is typical of fibrous PVDF materials, as can be seen in many works [41,42]. The layered S(PVDF+PA) sample showed the highest losses of 1.1% at 1% from all structures. It also exhibited high losses at low frequencies, where the dielectric loss was 6.6% at 1 Hz. The loss profile for this sample was similar to the PVDF fibrous sample, except that it was smoother.

### 3.2. Triboelectric Properties of Electrospun Samples

**Figure 6:**
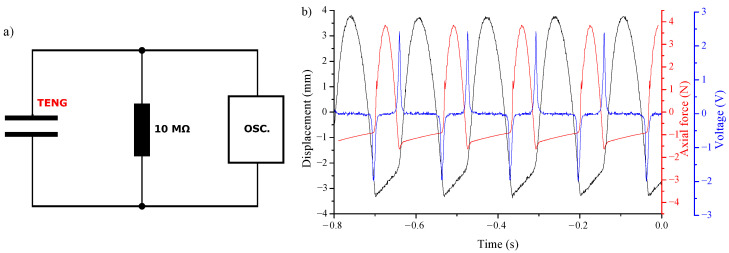
(**a**) Schematic of the measuring circuit. (**b**) Time record of the measurements on device D, where the output voltage, electrode displacement, and electrode contact force were recorded.

The fibrous structures were measured in a simple TENG operating in the CS mode. In the case of the TENG device, we monitored the output voltage of the TENG when the electrode spacing and the electrode contact force were also observed. An example of such an investigation is shown in Figure 6b, where device D was measured. The output voltage of TENG was measured by an oscilloscope with the internal resistance of 10 MΩ, as can be seen in Figure 6a. The aim was to see how these TENGs perform if they were used as simple dynamic force sensors. And to evaluate, if the fibrous composites provide any benefit in these applications.

**Figure 7:**
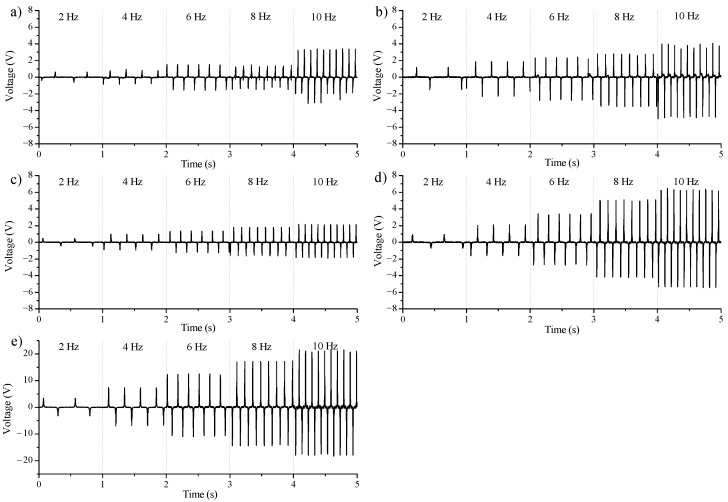
The output voltages for CS TENG at different excitation frequencies 2 Hz up to 10 Hz. The maximal gap between electrodes and force of touch of electrodes was constant. Individual TENGs are: (**a**) device A, (**b**) device B, (**c**) device C, (**d**) device D, and (**e**) device E.

Assembled TENG devices were tested at different excitation frequencies, where the force (approx. 10 N) and maximal displacement (approx. 9 mm) between electrodes were constant. The excitation frequency range was applied from 2 Hz up to 10 Hz. The output voltages for individual TENGs formed by a single dielectric (conductor-to-dielectric CS TENG), for different excitation frequencies, are shown in Figure 7, where (a) is for the TENG labeled as device A, (b) is for device B, (c) is for device C, and (d) is for device D. The Figure 7e shows the output voltage at different excitation frequencies for the device D which represents dielectric-to-dielectric CS TENG.

Device A exhibited a rather chaotic output voltage with a change in excitation frequency, with almost no difference between the 6 Hz and 8 Hz excitation frequencies (see Figure 7a). For the excitation frequency of 10 Hz, the output voltage jumped to approximately 6 V peak-to-peak. Device B showed a smooth increase in output voltage with increasing excitation frequency. For an excitation frequency of 10 Hz, the output voltage was approximately 8.7 V peak-to-peak (Figure 7b). Device C also showed a smooth increase in output voltage with increasing excitation frequency. The magnitude of the output voltage was approximately comparable to device A; however, it was much steadier as the excitation frequency changed. Device C showed an output voltage of around 2.1 V peak-to-peak at an excitation frequency of 10 Hz (Figure 7c). Surprisingly, device D exhibited the highest steepness of output voltage increase at increasing excitation frequency. The output voltage was relatively stable over time for each excitation frequency. This device D exhibited a peak-to-peak output voltage of 11.5 V at an excitation frequency of 10 Hz (Figure 7d).

In Figure 7e, it can be seen for the reference, how the output voltage behaved with increasing excitation frequency for the dielectric-to-dielectric CS TENG. It is clear to see that device E evidently generated the highest output voltage of the given TENG devices, where a peak voltage of 39 V was obtained at an excitation frequency of 10 Hz. However, device E represents an ideally assembled TENG when two materials with different triboelectric affinities are used.

**Figure 8:**
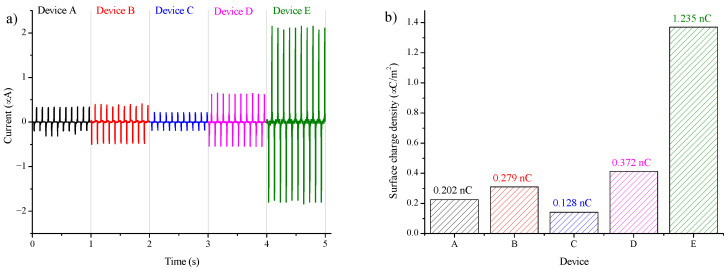
(**a**) The output current vs time for TENGs at excitation frequency 10 Hz. (**b**) The surface charge density of TENGs.

It is necessary to note that this study was not performed for reaching the maximal power output of TENGs. However, the surface charge density (SCD) was also calculated for complete information about our TENGs. From the voltage dependencies measured at a known electrical resistance, the current delivered by each TENG can be derived. The current versus time for all TENGs is shown in Figure 8a. This dependence was measured for the excitation frequency of 10 Hz. By integrating the electric current over time, we have obtained the amount of electric charge delivered by each device. By averaging all the maximum and minimum charge values, the average value of the charge generated by the TENG during contact and separation was calculated. As the TENG has a size of (3 × 3) cm^2^, the estimated surface charge density in the μC/m^2^ unit is able to calculate by dividing the averaged charge by the device size. The surface charge density of all TENGs is shown in Figure 8b. Between the conductor-to-dielectric TENGs, device D generated the highest current and thus achieved the highest SCD (*I*_peak-to-peak_ = 1.2 μA, *Q*_average_ = 0.372 nC). Followed by device B (*I*_peak-to-peak_ = 0.9 μA, *Q*_average_ = 0.279 nC), where the SCD value was approximately 1.3× lower than that of device D. Even slightly lower values were observed for device A (*I*_peak-to-peak_ = 0.6 μA, *Q*_average_ = 0.202 nC), and the lowest value was observed for device C (*I*_peak-to-peak_ = 0.4 μA, *Q*_average_ = 0.128 nC), where the SCD was 2.9× lower than device D. It can also be seen from Figure 8, that for a given excitation, device E (representing the dielectric-to-dielectric TENG) generated the highest current, and thus achieved the highest SCD (*I*_peak-to-peak_ = 3.9 μA, *Q*_average_ = 1.235 nC) of all the TENGs. The generated charge was more than three times higher than that of device D, which exhibited the highest SCD value among the conductor-to-dielectric TENGs.

We can see that the SCD values achieved are very low. However, as mentioned above, this study was not based on achieving the maximum power of the TENGs. It was a study of two frequently used materials in TENGs, where one has a strong triboelectric affinity for negative charge (PVDF) and the other for positive charge (PA). These materials were prepared both separately in fibrous form and together in a fibrous composite, where the fiber thicknesses and the thickness of the overall layer were maintained.

A summary of the experimental data and their mutual comparison based on the device type is given in Table 2. It shows the type of triboelectric device, the structure used in the triboelectric device, the dielectric constant, and the dielectric loss at 1 kHz. The last column of Table 2 demonstrates a simplified comparison of the TENG sensitivities to the excitation signal.

**Figure 9:**
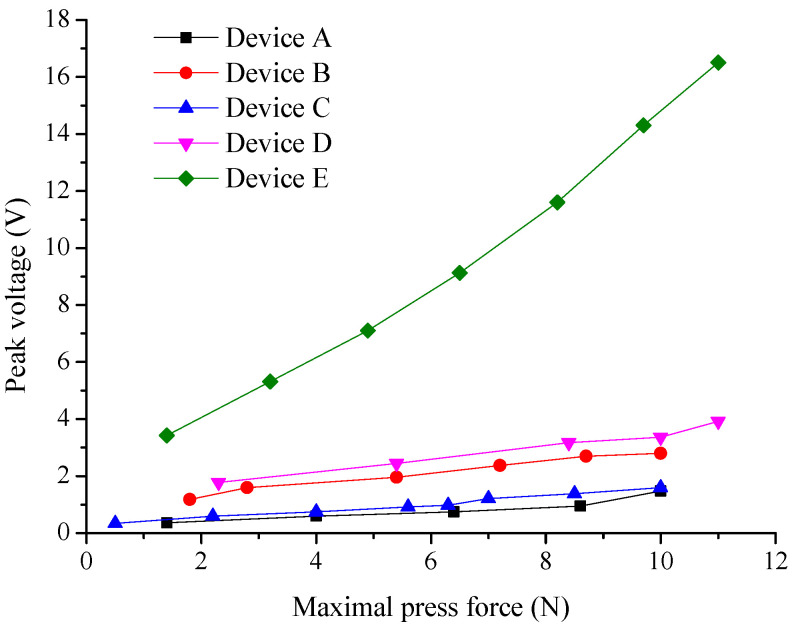
Peak voltage as a function of maximal press force for conductor-to-dielectric TENGs (device A, device B, device C and device D), and for dielectric-to-dielectric TENG, the device E.

In addition, TENG devices were tested in terms of contact compression force, where the compression force was gradually increased and the output voltage was recorded from the TENGs. The excitation frequency of the compression was the same for all TENGs at 6 Hz. The measurement result for our TENGs is shown in Figure 9. The increasing output voltage with increasing contact compression force for all TENGs was observed. In this measurement, the device with the lowest sensitivity was device A. Such a device generated the lowest output voltage of all the TENGs. Device C exhibited slightly higher output voltage values than device A, where the peak output voltage was always about 1.4× higher than that of device A. This was followed by a relatively large jump, where device B exhibited about 2.5× higher peak output voltage compared to device A. The highest sensitivity of all conductor-to-dielectric CS TENGs was observed for device D, where the output voltage reached approximately 3.4 V at peak at a compression force (10 N). Again, for comparison, device E is also shown here, where was observed the highest sensitivity to contact force of all the TENGs used. This is due to the full contact and separation of the two fibrous materials PVDF and PA occurring. The triboelectric charge density σ of device E is higher than that of devices A, B, C and D. According to Equations (1), (3) and (4), the higher triboelectric sensitivity of device E (dielectric-to-dielectric TENG) can be predicted compared to devices A, B, C and D (conductor-to-dielectric TENG).

A similar output voltage response was observed for device A (consisting of PA fibers) and device C (consisting of PVDF+PA intertwined fibers), which indicates that our PVDF fibers added no or only insignificant piezoelectric response. In the case of piezoelectrically active PVDF fibers, this device C would have to exhibit a higher voltage value than that observed in device A. Since this did not happen, it was concluded that mainly the triboelectric effect is applied here and the piezoelectric effect is suppressed.

This was confirmed by measuring the piezoelectric response on our PVDF fibers using the Berlincourt method, where PVDF fibers were sandwiched between two metal electrodes and measured by the $d_{33}$ meter. This measurement confirmed zero or negligible piezoelectric response (maximum 1 pC/N) on our PVDF fibers.

The findings of this investigation yielded interesting results concerning the structure of the fibrous composite of the S(PVDF+PA) sample. Device D, formed by this composite, exhibited the highest triboelectric response among all conductor-to-dielectric TENGs. Although device D contained two materials with different triboelectric affinities (PVDF and PA), this device generated the highest voltage in triboelectric measurements compared to device A, device B and device C. The findings are novel and provide an interesting insight into building an efficient TENG that consists of only one triboelectric layer. The most efficient TENG consists of two materials with different triboelectric affinities that are completely separated from each other before each contact, as seen in device E. Device E is shown here for comparison purposes only and serves as an ideal case of the most efficient TENG assembly. However, it is sometimes impossible to form a TENG with two triboelectric layers. Then, this study shows that by combining both PVDF and PA materials into a single triboelectric layer, a TENG can be constructed that generates a higher voltage than that TENG formed by a single layer from PVDF or PA material. Another interesting finding is that, simply combining the two materials by intertwining them into a single triboelectric layer is not effective, as seen in the results measured on device C. The resulting triboelectric layer must also contain a trapping layer, which is formed by the PVDF fibers in our device D. By further optimizing the thickness of this sink layer, it would be possible to achieve an even higher efficiency of this single-layer TENG. However, this study is beyond the scope of this paper.

## 4. Conclusions

The novelty of this work lies in the study of the triboelectric properties of a fibrous composite material that contains two materials which lie at different ends in a triboelectric series. This composite, contains interwoven fibers of both materials then forms a single triboelectric layer in a triboelectric device. It should also be pointed out that this study was not focused on the maximum power output of the TENG. The study was focused on the possibility of using these composite fibrous materials as an active sensor. Comparing their triboelectric properties and whether the composite structure has any benefits. The experiment demonstrated that, to achieve the highest possible triboelectric sensitivity and the possibility of using a triboelectric device in energy harvesting applications, the individual triboelectric materials must be separated entirely during release before their next contact. This was shown on device E, where PVDF fibers were used against PA fibers, where we reached peak voltage of the device 14.2 V at excitation force 10 N and frequency 6 Hz. If the materials were still in partial contact during release and subsequent compression, the device operates in a sliding mode rather than a contact separation mode. The sliding mode can be used here for sensing applications better than for energy harvesting. This was demonstrated for device C, where PVDF fibers were intertwined with PA fibers. This triboelectric device C showed slightly higher sensitivity to the excitation signal than device A, which consisted of PA fibers. Device C generated the peak voltage of 1.6 V compared to device A, where the generated peak voltage was 1.5 V. Device A exhibited the lowest excitation signal sensitivity of all the assembled devices. Device D, which used a layered fibrous structure, showed the highest excitation signal sensitivity compared to the prepared conductor-to-dielectric triboelectric devices, where the measured voltage response was 3.5 V at peak. Such an improvement of the triboelectric response may be due to forming the trapping layer consisting of PVDF fibers placed between the electrode and the combined PVDF and PA layer. This device D exhibited 3.5× higher sensitivity to the excitation signal than device B composed of PVDF fibers, where the peak voltage 2.8 V was measured. The proposed study provides an interesting perspective on the fabrication of TENG formed by a single dielectric, where this dielectric contains a combination of two materials with different triboelectric affinities.

## Figures and Tables

**Table 1:** Overview of samples and processing parameters.

Sample	Structure	Needle (–)	Rotation Speed (rpm)
PA	PA fibers	19G	300
PVDF	PVDF fibers	17G	300
PVDF+PA	PVDF+PA intertwined fibers	17G/19G	300
S(PVDF+PA)	PVDF//PVDF+PA//PA *	see above	2000

* Please note that the double slash used here is used to distinguish individual layers in the stacked structure.

**Table 2:** Summary of the experimental data.

Device	Type of the Triboelectric Device	Fiber Dielectric Sample	*ε*_r_ at 1 kHz	tan *δ* (×10^3^) at 1 kHz	Peak Voltage (*V*) at *F* = 10 N, *f* = 6 Hz	Triboelectric Response
A	Conductor-to-dielectric	PA	1.19	7.44	1.5	Lowest
B		PVDF	1.57	6.68	2.8	Medium
C		PVDF+PA	1.24	7.17	1.6	Low
D		S(PVDF+PA)	1.45	10.56	3.5	High
E	Dielectric-to-dielectric	PVDF opposite PA	1.57 PVDF	6.68 PVDF	14.2	Highest
			1.19 PA	7.44 PA		

Note: Thickness of the dielectric layer was 20 μm.

## Sample Availability

Samples are available on demand from Pavel Tofel. E-mail: tofel@vut.cz.

## Abbreviations

The following abbreviations are used in this manuscript:

AA	Acetic acid
Ac	Acetone
CS	Contact-separation
DMSO	Dimethylsulphoxide
FA	Formic acid
FT	Freestanding triboelectric-layer
LS	Lateral sliding
PA	Polyamide 6
PDMS	Polydimethylsiloxane
PVDF	Polyvinylidene fluoride
SE	Single-electrode
SEM	Scanning electron microscopy
SCD	Surface charge density
